# Economic evaluation of critically ill adult CAR-T cell recipients—analysis from a healthcare payer perspective

**DOI:** 10.1007/s00063-024-01230-z

**Published:** 2024-12-30

**Authors:** Kevin Roedl, Paymon Ahmadi, Sonja Essmann, Sarosh Aamir, Markus Haar, Francis Ayuk, Panagiotis Karagiannis, Nicolaus Kröger, Stefan Kluge, Dominic Wichmann

**Affiliations:** 1 Department of Intensive Care Medicine, University Medical Centre Hamburg-Eppendorf, Martinistraße 52, 20246 Hamburg, Germany; 2 Department of Stem Cell Transplantation, University Medical Centre Hamburg-Eppendorf, Hamburg, Germany; 3 Department of Haematology, Oncology and Bone Marrow Transplantation with Section of Pneumology, University Medical Centre Hamburg-Eppendorf, Hamburg, Germany

**Keywords:** Intensive care unit, Critical illness, Chimeric antigen receptor T, Lymphoma, Costs and cost analysis, Intensivstation, Kritische Krankheit, Chimärer Antigenrezeptor T, Lymphom, Kosten und Kostenanalyse

## Abstract

**Background:**

CAR-T cell (chimeric antigen receptor T) therapy is now part of standard of care treatment of B‑cell lineage malignancies. Although it is an effective treatment, it comes along with adverse side effects and toxicities that may require intensive care therapy. The costs related to critical care therapy in critically ill patients after CAR‑T administration have not been evaluated.

**Patients and methods:**

Retrospective analysis of all patients who had received CAR‑T therapy and were admitted to the intensive care unit (ICU) of a tertiary care university medical centre in Germany between 1 January 2019 and 31 December 2022. Cause of admission and ICU therapy as well as treatment and total hospitals costs were evaluated.

**Results:**

Thirty patients with a history of CAR-T cell therapy for underlying haematological malignancy were included. The median age of all patients was 60 years (interquartile range [IQR] 50–70) and 37% (*n* = 11) were female. 93% (*n* = 28) of patients had non-Hodgkin lymphoma and 7% (*n* = 2) had multiple myeloma. The cohort was stratified whether the ICU admission was CAR‑T therapy related (i.e. within 30 days after CAR‑T therapy; 73%, *n* = 22) or the admission was of an other cause (> 30 days after CAR‑T therapy) (27%, *n* = 8). The median duration from CAR‑T therapy to ICU admission was 6 (range 5–8) days in CAR-T cell therapy associated ICU admissions compared with 52 (range 31–126) days in other admissions. The overall illness severity on admission was numerically higher in CAR-T-related ICU admission compared to other admissions (46 vs. 43 points, *p* = 0.18). Vasopressor therapy (50% vs. 75%; *p* = 0.19), invasive mechanical ventilation (27% vs. 50%; *p* = 0.24) and renal replacement therapy (14% vs. 50%; *p* < 0.05) were used in CAR-T-associated admission compared to other admissions, respectively. The ICU mortality (23% vs. 50%; *p* = 0.15) was higher in patients with other ICU admission. Median total costs of the entire inpatient stay in hospital were € 27,845 (range 8661–368,286 €) in CAR-T-associated ICU admissions compared to € 59,234 (range 23,182–127,044 €) in the group of other ICU admissions (costs of the CAR‑T product not included).

**Conclusion:**

In relation to the total costs of CAR-T-cell therapy (production of the CAR‑T product), therapy-associated complications have a relatively low impact on the costs and utilization of ICU resources.

**Supplementary Information:**

The online version of this article (10.1007/s00063-024-01230-z) contains supplementary material, which is available to authorized users.

## Abstract (de)

**Hintergrund:**

Die Therapie mit CAR-T-Zellen (chimäre Antigenrezeptor-T-Zellen) gehört heute zur Standardbehandlung maligner Erkrankungen der B‑Zell-Linie. Obwohl es sich um eine wirksame Therapie handelt, geht sie mit unerwünschten Nebenwirkungen und Toxizitäten einher, die eine intensivmedizinische Behandlung erfordern können. Die Kosten für die Intensivtherapie bei schwerkranken Patienten nach einer CAR-T-Gabe sind nicht untersucht worden.

**Patienten und Methoden:**

Es wurde eine retrospektive Analyse aller Patienten durchgeführt, die eine CAR-T-Therapie erhalten hatten und zwischen dem 1. Januar 2019 und dem 31. Dezember 2022 auf der Intensivstation (ICU) eines Universitätsklinikums in Deutschland aufgenommen wurden. Ausgewertet wurden der Aufnahmegrund, die speziellen intensivmedizinischen Maßnahmen sowie die Behandlungs- und Gesamtkosten im Krankenhaus.

**Ergebnisse:**

In die Studie wurden 30 Patienten aufgenommen, die wegen einer hämatologischen Malignität eine CAR-T-Zell-Therapie erhalten hatten. Das Durchschnittsalter aller Patienten betrug 60 Jahre (Interquartilsbereich [IQR] 50–70), und 37% (*n* = 11) waren weiblich. Insgesamt hatten 93% (n=28) der Patienten ein Non-Hodgkin-Lymphom und 7% (*n* = 2) ein multiples Myelom. Die Kohorte wurde danach stratifiziert, ob die Aufnahme auf der ICU im Zusammenhang mit der CAR-T-Therapie erfolgte (d. h. innerhalb von 30 Tagen nach der CAR-T-Therapie; 73%, *n* = 22) oder ob die Aufnahme aus anderen Gründen mehr als 30 Tage nach der CAR-T-Therapie erfolgte (27%, *n* = 8). Die mediane Dauer von der CAR-T-Therapie bis zur Aufnahme auf die ICU betrug 6 (Range: 5–8) Tage bei den CAR-T-Zell-Therapie-assoziierten Einweisungen im Vergleich zu 52 (Range: 31–126) Tagen bei Einweisungen aus anderen Gründen. Der Gesamtschweregrad der Erkrankung bei Aufnahme war bei CAR-T-assoziierten Intensivstationen numerisch höher als bei Aufnahmen aus anderen Gründen (46 vs. 43 Punkte; p=0,18). Bei CAR-T-assoziierten Aufnahmen kamen im Vergleich zu anderen Einweisungen Vasopressoren (50% vs. 75%; *p* = 0,19), invasive mechanische Beatmung (27% vs. 50%; p=0,24) und Nierenersatztherapie (14% vs. 50%; p<0,05) zum Einsatz. Die Mortalität auf der ICU (23% vs. 50%; *p* = 0,15) war höher bei Patienten mit Einweisungen aus anderen Gründen. Die medianen Gesamtkosten des gesamten stationären Aufenthalts im Krankenhaus betrugen 27.845 € (Range: 8661–368.286 €) bei CAR-T-assoziierten ICU-Aufnahmen im Vergleich zu 59.234 € (Range: 23.182–127.044 €) in der Gruppe der ICU-Aufnahmen aus anderen Gründen (Kosten für das CAR-T-Produkt nicht eingeschlossen).

**Schlussfolgerung:**

Im Verhältnis zu den Gesamtkosten der CAR-T-Zell-Therapie (Herstellung des CAR-T-Produkts) haben therapieassoziierte Komplikationen einen relativ geringen Einfluss auf die Kosten und die Inanspruchnahme von Ressourcen der Intensivstation.

## Background

CAR-T cell (chimeric antigen receptor T) therapy has emerged in recent years as very effective treatment in relapsed/refractory B‑cell malignancies [1–4]. This immunotherapy is based on the genetic modification of autologous cytotoxic T lymphocytes and the recognition of a tumour-associated cell surface antigen through the CAR resulting in tumour lysis [4]. CAR-T cell therapy is associated with high costs especially due to the complex manufacturing process.

Potent therapies, such as CAR-Ts, come along with potent adverse effects and toxicities [5]. Critical care management plays an important role in patients receiving CAR‑T, where up to half of the patients might need admission to the ICU and lifesaving interventions [5–7]. Cytokine release syndrome (CRS) and immune effector cell-associated neurotoxicity syndrome (ICANS) represent the two most frequent life-threatening adverse events directly associated with CAR-T cell therapy [5]. The incidence of CRS is reported to be between 58 and 93%, depending on the underlying haematologic malignancy, tumour burden as well as the type of CAR‑T [1, 2, 8–10]. Typically, the onset of CRS occurs within the first days after CAR‑T. CRS can be very severe, resulting in multiple organ failure with need for ICU admission [11, 12]. ICANS represents the second specific complication of CAR-T cell therapy occurring in 20–60% of patients [1–3, 13]. It occurs in a median time of 4–10 days after CAR-T cell infusion and rarely occurs in the absence of CRS [11]. The clinical symptoms of ICANS are extremely diverse. Besides these two quite common complications, the immunocompromised state poses a great risk for opportunistic infections, further complicated by septic shock, thereby, requiring critical care treatment [11, 14].

To date, various studies assessed the additional costs including lymphodepletion, outpatient visits and hospitalization, apart from acquisition and infusion of CAR T‑cells [15]. However, the costs related to critical care therapy in critically ill patients after CAR‑T administration have not been evaluated. The aim of the present study was to evaluate causes and costs of critical care therapy in CAR‑T recipients.

## Methods

### Study design, setting and ethics

Data of all adult patients who underwent CAR-T cell therapy and were admitted to the Department of Intensive Care Medicine at the University Medical Centre Hamburg-Eppendorf (Germany) between January 1, 2019 and December 31, 2022 were analysed. The last date of follow-up was October 31, 2023. The department currently comprises 12 intensive care wards and cares for all critically ill adult patients of the hospital with a total capacity of 140 beds. The Ethics Committee of the Hamburg Chamber of Physicians was informed about the study (No. 2023-300294-WF). Due to the retrospective nature of the study and the de-identified study data, the need for explicit informed consent was waived in accordance with § 12 of the Hamburg Hospital Law. The study was in accordance with the ethical standards of the Ethics Committee of the Hamburg Chamber of Physicians on human experimentation (regional) and with the Helsinki Declaration of 1975, as most recently amended.

### Inclusion and exclusion criteria

All consecutive adult patients who received CAR-T cell therapy and were admitted to the ICU were included in the study. All patients below 18 years of age, patients without CAR-T cell therapy admitted to the ICU, or patients with incomplete clinical data were excluded.

### Data collection

Data were collected through the electronical patient data management system (PDMS, Integrated Care Manager® [ICM], version 9.1; Draeger Medical, Luebeck, Germany). The extracted data included age, gender, comorbidities, primary admission diagnosis, length of ICU- and hospital-stay, total inpatient reimbursement data, outcome, treatment modalities and organ support (mechanical ventilation, vasopressor, renal replacement therapy, blood transfusions) during the ICU stay. Furthermore, oncologic disease and treatment modality data were assessed.

### Study definitions and patient management

Patients were categorised based on the relation of their ICU admission diagnosis to CAR-T cell therapy (CRS and ICANS) and generally occurring within the first 30 days after CAR‑T therapy or conditions not directly related to CAR-T cell therapy (e.g. infection, sepsis, haemodynamic instability) referred as other admissions. The severity of illness was evaluated by sequential organ failure assessment (SOFA) [16] and simplified acute physiology (SAPS II) [17] score on ICU admission. The Charlson Comorbidity Index (CCI) [18] was calculated in all patients. Sepsis and septic shock were defined according to the 2016 Third International Consensus Definition for Sepsis and Septic Shock [19]. CRS and ICANS were graded according to international consensus criteria [20].

All relevant economic parameters were assessed, focusing on reimbursement of diagnosis-related group (DRG) flat rates. DRG costs are the mathematical products of the DRG case-mix and the base rate (BR) for the state of Hamburg (Germany). The average BR during the study period (2016–2022) was € 3571 (€ 3350–3830) [21]. Total inpatient reimbursement data were determined using codes from the DRG, the new diagnostic and treatment methods regulation (NUB), and additional charges “Zusatzentgelte” (ZE).

### Cost analysis

Data for the cost analysis were extracted from the enterprise resource planning software (SAP Software, SAP AG 2022, Walldorf, Germany) of the University Medical Centre Hamburg-Eppendorf (UKE), including billing data. Administrative data were collected in the form of length of hospital stay (LOS), ICD codes, OPS codes, G‑DRG (German Diagnosis-Related Group) codes, G‑DRG case mix, invasive ventilation, date of inpatient admission, date of discharge and case type. The G‑DRG reimbursement system classifies inpatient treatments according to the underlying disease and resource consumption. The system assigns a specific DRG code to each patient based on their diagnosis (ICD codes), treatment procedures (OPS codes), and further individual factors such as age and LOS. Using the DRG system, a detailed profile of the costs associated with the treatment was obtained from a German healthcare payers perspective. Hospitalization reimbursement data were based on the G‑DRG system that is regulated by the Institute for the Hospital Remuneration System (InEK GmbH, Siegburg, Germany) [22].

### Statistical analysis

Data are presented as absolute numbers and relative frequency or median and with interquartile range (IQR). Categorial variables were compared via Chi-square analysis or Fisher’s exact test, as appropriate. Continuous variables were compared via Mann–Whitney U‑test. We clinically assessed factors associated with mortality.

Statistical analysis was conducted using IBM SPSS Statistics (version 24.0, IBM Corp., Armonk, NY, USA). No correction for multiple hypothesis testing was applied. Generally, a *p*-value < 0.05 was considered statistically significant. The study was prepared in accordance with the STROBE (STrengthening the Reporting of OBservational studies in Epidemiology) recommendations.

## Results

During the study period from 01 January 2019 to 31 December 2023 we identified 30 patients with history of CAR-T cell therapy and underlying haematological disease who were admitted to one of the ICUs of the Department of Intensive Care Medicine of the University Medical Centre Hamburg-Eppendorf. All patients were included in the present study; no patients were excluded due to incomplete data.

### Baseline characteristics

The baseline characteristics of the cohort are shown in Table 1. The study population had a median age of 60 (IQR 50–70) years; 37% (*n* = 11) were female. The median weight and height were 77.2 (68.3–85) kg and 174 (166–185) cm, respectively. The median body mass index (BMI) was 24.9 (22.5–28.1) m^2^/kg. All patients had an underlying haematologic disease. In all, 93% (*n* = 28) of patients had non-Hodgkin lymphoma and 7% (*n* = 2) had multiple myeloma. The patients were admitted to the ICU from the stem-cell transplantation unit (90%, *n* = 27), normal ward (7%, *n* = 2) and the emergency department (3%, *n* = 1), respectively. The median Charlson Comorbidity Index (CCI) was 4 (4–5) points and the median SAPS II score was 45 (38–48) points at ICU admission. Overall, 57% (*n* = 17) required vasopressors, 33% (*n* = 10) received non-invasive or invasive mechanical ventilation and 23% (*n* = 7) received renal replacement therapy. The median duration of ICU and hospital stay was 3.0 (1.3–10.8) and 33.5 (28.8–43.5) days. The ICU mortality was 30% (*n* = 9).

**Table 1:** Baseline characteristics of all patients with history of CAR‑T therapy and ICU admission

Variables	All patients (*n* = 30)
Age (years)	60 (50–70)
Weight (kg)	77.2 (68.3–85)
Height (cm)	174 (166–185)
BMI (m^2^/kg)	24.9 (22.5–28.1)
Female	11 (37)
**Oncologic disease characteristics**	
*Underlying disease*	
Non-Hodgkin lymphoma	28 (93)
Multiple myeloma	2 (7)
*Duration from CAR‑T administration to ICU admission (days)*	8 (5–24)
**Admission from**	
Stem-cell transplantation unit	27 (90)
Emergency department	1 (3)
Normal ward	2 (7)
**Disease severity**	
Charlson comorbidity index, points	4 (4–5)
SAPS II—admission (points)	45 (38–48)
**Procedures/therapies**	
*Vasopressors*	17 (57)
*Mechanical ventilation*	10 (33)
Non-invasive	6 (20)
Invasive	0 (33)
*Duration of IMV (days)*	3.9 (2.5–13.4)
*Renal replacement therapy*	7 (23)
*Tracheostomy*	2 (7)
**Outcome**	
Duration ICU stay (days)	3.0 (1.3–10.8)
Duration hospital stay (days)	33.5 (28.8–43.5)
Died in ICU	9 (30)
Died in hospital	10 (33)

Data are expressed as *n* (%) or median (interquartile range—IQR 25/75%)

*kg* kilogram, *cm* centimetre, *SAPS* simplified acute physiology score, *IMV* invasive mechanical ventilation, *ICU* intensive care unit

### Clinical characteristics depending on the reason for admission (CAR-T-related or other)

The cohort was divided into two groups according to the cause of admission. In 73% (*n* = 22) of patients the ICU admission was directly associated with CAR‑T therapy. Reasons for CAR‑T therapy-associated ICU admission were CRS (59%, *n* = 13) and/or ICANS (54%, *n* = 12). Patients admitted due to other causes had respiratory insufficiency (63%, *n* = 5), sepsis (13%, *n* = 1), acute abdomen (13%, *n* = 1), and miscellaneous admission cause (13%, *n* = 1). Detailed characteristics of patients including baseline characteristics and treatment modalities are shown in Table 2.

**Table 2:** Characteristics of patients with CAR-T-associated ICU admission and other causes for ICU admission

Variables	CAR-T-associated ICU admission (*n* = 22)	Other ICU admission (*n* = 8)	*p*-value
Age (years)	63 (58–75)	46 (39–57)	< 0.001
Weight (kg)	77.2 (68.9–85.0)	78.6 (61.8–88.4)	0.91
Height (cm)	173 (166–180)	184 (171–186)	0.19
Female	9 (41)	2 (25)	0.42
**Oncologic disease characteristics**			
*Underlying disease*			
Non-Hodgkin lymphoma	20 (91)	8 (100)	0.38
Multiple myeloma	2 (9)	0 (0)	0.53
*Duration from CAR‑T administration to ICU admission (*days)	6 (5–8)	52 (31–126)	< 0.001
**Disease severity**			
Charlson comorbidity index, points	4 (4–5)	4 (4.3–5)	0.63
SAPS II—admission (points)	46 (41–50)	43 (36–45)	0.18
**Procedures/therapies—ICU**			
Vasopressors	11 (50)	6 (75)	0.19
Invasive MV	6 (27)	4 (50)	0.24
Duration of MV (days)	3.9 (1.5–23.8)	3.5 (2.3–4.3)	0.99
Renal replacement therapy	3 (14)	4 (50)	< 0.05
Tracheostomy	2 (9)	0 (0)	0.38
**Complications—ICU stay**			
*Pneumonia*	4 (18)	6 (75)	< 0.05
*ARDS*	3 (14)	5 (63)	< 0.05
Mild	0 (0)	2 (25)	–
Moderate	1 (18)	0 (0)	–
Severe	2 (9)	3 (38)	–
*Pulmonary embolism*	0 (0)	0 (0)	–
*Cardiac arrest*	3 (14)	0 (0)	0.38
*Myocardial infarction*	0 (0)	0 (0)	–
*Septic shock*	5 (23)	6 (75)	< 0.001
**Outcome**			
Duration ICU stay (days)	3 (2–9)	38.5 (33–59)	0.80
Duration hospital stay (days)	33 (27–41)	7 (1–18.5)	0.22
Died in ICU	5 (23)	4 (50)	0.15
Died in hospital	5 (23)	5 (63)	< 0.05

Data are expressed as *n* (%) or median (interquartile range—IQR 25/75%)

*kg* kilogram, *cm* centimetre, *SAPS* simplified acute physiology score, *IMV* invasive mechanical ventilation, *ICU* intensive care unit

Age was higher amongst patients with CAR-T-associated ICU admission (63 vs. 46 years; *p* < 0.001). Further demographic characteristics including gender, weight and height were comparable between both groups. Patients with CAR-T-associated ICU admission had an underlying non-Hodgkin lymphoma (91%, *n* = 20) or multiple myeloma (9%, *n* = 2). All patients with non-CAR-T-associated ICU admission had non-Hodgkin lymphoma (100%, *n* = 8). The median duration from CAR‑T therapy to ICU admission was 6 (5–8) days in associated ICU admissions compared with 52 (31–126) days in admissions due to other causes. The median CCI (4 vs. 4, *p* = 0.63) and SAPS II (46 vs. 43 points, *p* = 0.18) on admission were numerically higher in patients with CAR-T-associated ICU admission. Detailed characteristics on comorbidities are shown in Supplementary Table 1.

Vasopressor therapy was necessary in 50% (*n* = 11) and 75% (*n* = 6), respectively (*p* = 0.19). Invasive mechanical ventilation was necessary in 27% (*n* = 6) and 50% (*n* = 4), with a median duration of 3.9 (1.5–23.8) and 3.5 (2.3–4.3) days in both groups (*p* = 0.24 and 0.99). Renal replacement therapy had to be initiated in 14% (*n* = 3) and 50% (*n* = 4) patients, respectively (*p* < 0.05). Complications during the ICU stay were more frequent in patients with other admission causes and are detailed displayed in Table 2. The duration of ICU and hospital stay was longer in patients with other causes for ICU admission. The ICU mortality (23% vs. 50%, *p* = 0.15) and the hospital mortality (23% vs. 63%, *p* < 0.05) were higher in patients with other causes for ICU admission.

### Economic evaluation

**Fig. 1:**
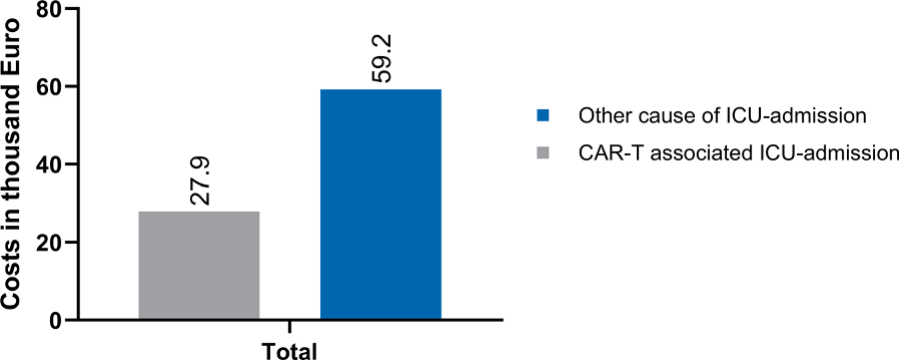
Median total hospital charges (excluding CAR‑T costs) for patients with history of CAR‑T therapy and other cause of intensive care unit (ICU) admission. CAR‑T chimeric antigen receptor T

Table 3 presents median values, ranges and percentages of reimbursement-relevant key figures. The median hospital length of stay in CAR-T-associated ICU admissions was 33.5 (13–201) compared with 39.5 (23–79) days in other ICU admissions. The median ICU length of stay was 80 (12–1988) compared with 174 (7–612) h. Invasive mechanical ventilation was required for a median of 0 (0–683) and 43 (0–364) h. The median total costs of the total inpatient stay in hospital (sum of DRG flat rates, NUB and ZE charges) were € 27,845 (8661–368,286) in CAR-T-associated ICU admissions and € 59,234 (23,182–127,044) in the group of other causes for ICU admissions. See also Fig. 1.

**Table 3:** Reimbursement relevant key-figures

Parameters	CAR-T-associated ICU admission (*n* = 22)	Other ICU admission (*n* = 8)
Length of hospital stay	33.5 (13–201)	39.5 (23–79)
ICU hours (range)	80 (12–1988)	174 (7–612)
Proportion of ICU stay on complete LOS (%)	8.1 (1.8–77.8)	14.9 (0.9–66.5)
Invasive ventilation hours (range)	0 (0–683)	43 (0–364)
Total hospital charges in € (without costs of CAR-T)	27,845 (8661–368,286)	59,234 (23,182–127,044)

Data are expressed as *n* (%) or median (interquartile range—IQR 25/75%)

*LOS* length of stay, *ICU* intensive care unit

## Discussion

CAR-T cell therapy is a financially demanding treatment, prompting an examination of the cost–benefit ratio at the population and patient levels. Beyond direct CAR‑T therapy, the utilization of ICU resources and subsequent follow-up costs may be significant. To our knowledge this is the first study to analysing the financial implications of ICU admission of recipients of CAR-T cell therapy in the German healthcare system based on the reimbursement regulations, allowing us to elucidate the precise economic impact on the healthcare payer. A key advantage of the health economic approach is its ability to provide quick and accurate assessments of the actual financial burden on health insurers, based on underlying billing information. When compared to patients admitted to the ICU for other conditions not directly associated with CAR-T cell therapy, costs were found to be relatively low (€ 27,845 vs. € 59,234) and resource utilization was modest, also relative to the total cost including the CAR-T cell product.

A comparison of the costs of CAR‑T therapy with other therapy options for these disease stages (bidirectional antibodies, antibody–conjugated drugs) shows that CAR‑T therapy, in addition to the aspect of long-term therapeutic success, can also be assessed positively from a financial perspective [23]. Bispecific antibodies like mosunetuzumab and teclistamab have gained approval for certain lymphomas and multiple myeloma, with new candidates like epcoritamab and glofitamab anticipated for approval. Comparing these bidirectional antibodies against the CAR‑T therapy is challenging as efficacy and durability are still being evaluated [24].

In this study, we sought to categorize the reimbursements into distinct cost-centre groups (e.g. ward, intensive care, operating rooms, radiology, laboratories) and cost category groups (e.g. labor costs, material costs, infrastructure costs). This approach aligns with the DRG system framework, where the InEK cost-matrix classifies the financial aspects of hospital into specific sectors.

Conventionally, hospitals receive additional remuneration for inpatient stays exceeding the average duration, in the form of surcharges. Nevertheless, it is pertinent to note that these surcharges, alongside supplementary fees denoted as “Zusatzentgelte” (ZE) and “Neue Untersuchungs- und Behandlungsmethoden” (NUB), cannot be allocated using the InEK cost-matrix, thus limiting the ability to make an isolated statement about the ICU reimbursement. Consequently, this study provides a detailed analysis of the costs from the healthcare payer’s perspective for the entire hospital case and it highlights that intensive care was a major cost driver for these patients, to the extent that 10% of total inpatient time was spent in the ICU.

This study has several limitations. First, we show the results of a retrospective single centre study and small sample size. Conclusions should therefore be made with caution. Second, residual confounding from unmeasured covariables cannot be entirely excluded. Third, depending on the reimbursement regulations the analysis may result in different outcomes when performed in different healthcare systems.

## Conclusion

Intensive care unit (ICU) costs are relatively low in relation to the total costs of chimeric antigen receptor T (CAR-T) cell therapy.

## Supplementary Information

**Supplementary Table 1**: Pre-existing comorbidities of patients with CAR-T-associated ICU admission and other causes for ICU admission

## Data Availability

The datasets supporting the conclusions of this article are included within the article.

## Declarations

The Ethics Committee of the Hamburg Chamber of Physicians was informed about the study (No. 2023-300294-WF). Due to the retrospective nature of the study and the deidentified study data, the need for explicit informed consent was waived. The study was in accordance with the ethical standards of the Ethics Committee of the Hamburg Chamber of Physicians on human experimentation (regional) and with the Helsinki Declaration of 1975, as most recently amended.

The supplement containing this article is not sponsored by industry.
