# Supplementary material for: Economic evaluation of critically ill adult CAR-T cell recipients—analysis from a healthcare payer perspective
Source: Med Klin Intensivmed Notfmed. 2024 Dec 30;120(Suppl 1):1–7. doi: 10.1007/s00063-024-01230-z (PMC12708791; doi:10.1007/s00063-024-01230-z)
Supplement: Supplementary file 1 — Supplementary Table 1: Pre-existing comorbidities of patients with CAR-T-associated ICU admission and other causes for ICU admission [file 63_2024_1230_MOESM1_ESM.docx]

Supplementary Table 1 – Pre-existing comorbidities of patients with CAR-T associated ICU admission and other causes for ICU-admission

| *Parameters* | *CAR-T associated ICU-admission*  *(n = 22)* | *Other ICU-admission*  *(n = 8)* | *p*-value |
| --- | --- | --- | --- |
| Cerebral arterial disease *n (%)* | 4 (18) | 0 (0) | 0.27 |
| Chronic lung disease *n (%)* | 1 (5) | 1 (13) | 0.47 |
| Chronic kidney disease *n (%)* | 1 (5) | 1 (13) | 0.47 |
| Congestive heart disease *n (%)* | 2 (9) | 0 (0) | 0.53 |
| Connective tissue disease *n (%)* | 0 (0) | 0 (0) | - |
| Coronary heart disease *n (%)* | 1 (5) | 0 (0) | 0.73 |
| Dementia *n (%)* | 0 (0) | 0 (0) | - |
| Diabetes Mellitus *n (%)* | 3 (14) | 0 (0) | 0.38 |
| Liver cirrhosis *n (%)* | 1 (5) | 0 (0) | 0.73 |
| Peripheral arterial disease *n (%)* | 0 (0) | 0 (0) | - |
